# Detection of neurophysiological markers of cognitive reserve: an EEG study

**DOI:** 10.3389/fnagi.2024.1401818

**Published:** 2024-08-07

**Authors:** Osamu Katayama, Yaakov Stern, Christian Habeck, Annabell Coors, Sangyoon Lee, Kenji Harada, Keitaro Makino, Kouki Tomida, Masanori Morikawa, Ryo Yamaguchi, Chiharu Nishijima, Yuka Misu, Kazuya Fujii, Takayuki Kodama, Hiroyuki Shimada

**Affiliations:** ^1^Department of Preventive Gerontology, Center for Gerontology and Social Science, National Center for Geriatrics and Gerontology, Obu, Aichi, Japan; ^2^Japan Society for the Promotion of Science, Chiyoda-ku, Tokyo, Japan; ^3^Department of Physical Therapy, Graduate School of Health Sciences, Kyoto Tachibana University, Oyake, Yamashina-ku, Kyoto, Japan; ^4^Columbia University Vagelos College of Physicians and Surgeons, New York, NY, United States

**Keywords:** cognitive reserve, electroencephalography, dorsal attention network, ventral attention network, neurophysiological markers, community-dwelling older adults

## Abstract

**Background and objectives:**

Cognitive reserve (CR) is a property of the brain that allows for better–than–expected cognitive performance relative to the degree of brain change over the course of life. However, neurophysiological markers of CR remain under-investigated. Electroencephalography (EEG) features may function as suitable neurophysiological markers of CR. To assess this, we investigated whether the dorsal attention network (DAN) and ventral attention network (VAN) activities, as measured during resting–state EEG, moderate the relationship between hippocampal volume and episodic memory.

**Methods:**

Participants were recruited as part of the National Center for Geriatrics and Gerontology–Study of Geriatric Syndromes. Hippocampal volume was determined using magnetic MRI, and episodic memory was measured using word lists. After testing the effect of hippocampal volume on memory performance using multiple regression analysis, we evaluated the interactions between hippocampal volume and DAN and VAN network activities. We further used the Johnson–Neyman technique to quantify the moderating effects of DAN and VAN network activities on the relationship between hippocampal volume and word list memory, as well as to identify specific ranges of DAN and VAN network activity with significant hippocampal–memory association.

**Results:**

A total of 449 participants were included in this study. Our analysis revealed significant moderation of DAN with a slope of β = −0.00012 (95% CI: −0.00024; −0.00001, *p* = 0.040), and VAN with a slope of β = 0.00014 (95% CI: 0.00001; 0.00026, *p* = 0.031). Further, we found that a larger hippocampal volume was associated with improved memory performance, and that this association became stronger as the DAN activity decreased until a limit of DAN activity of 944.9, after which the hippocampal volume was no longer significantly related to word-list memory performance. For the VAN, we found that a higher hippocampal volume was more strongly associated with better memory performance when VAN activity was higher. However, when VAN activity extended beyond −914.6, the hippocampal volume was no longer significantly associated with word-list memory.

**Discussion:**

Our results suggest that attentional networks help to maintain memory performance in the face of age-related structural decline, meeting the criteria for the neural implementation of cognitive reserve.

## 1 Introduction

Cognitive reserve (CR) is a property of the brain that allows for better cognitive performance than could be expected given the degree of life–course brain changes, brain injury, or disease (Stern et al., 2023). Typically, measures such as childhood intelligence quotient (IQ) (Deary et al., 2004), educational history (Bennett et al., 2003), and occupational exposure (Habeck et al., 2019) have been examined as proxy markers of CR (Song et al., 2022), as research has shown that they are associated with improved cognitive performance, given the same degree of age–related brain changes (Stern et al., 2023). Overall, higher CR may delay cognitive decline and the onset of dementia. However, investigation of proxy CR markers such as childhood IQ, educational history, and occupational exposure, do not provide information on the neural implementation of CR. Therefore, in the present study, we investigated whether differential expression of the dorsal attention network (DAN) and ventral attention network (VAN) could be involved in CR by moderating the impact of differences in hippocampal volume on memory performance.

Prior research has established a close relationship between the hippocampal volume and memory function (Laakso et al., 2000), while hippocampal atrophy has been shown to be associated with decreased verbal memory capacity (Walhovd et al., 2004). A decline in episodic memory is the most common cognitive function-related symptom in patients diagnosed with Alzheimer’s disease (AD) (Albert, 2011). In mild cognitive impairment (MCI), which is considered as a transitional period between healthy aging and AD dementia, the subcategory of patients with impaired memory function (amnestic MCI) has been shown to be at a high risk of transitioning to AD (Petersen et al., 2001). As such, the assessment of both immediate and delayed episodic memory is important for the early detection of cognitive decline (Hamel et al., 2015).

Many studies have investigated brain activation patterns using resting–state (rs) functional MRI (fMRI). Further, an increasing number of reports have investigated statistical differences in the activity of the dorsal attention network (DAN) and ventral attention network (VAN) among healthy subjects, subjects with MCI, and patients with AD, using rs–fMRI (Zhang et al., 2015; Wu et al., 2022). Further, the trajectory of an individual’s brain network composition over time, as measured by fMRI, has been shown to be independent of AD–related genetic risk factors (APOE status), AD–related pathology (cerebrospinal fluid phosphorylated tau, and cortical amyloid), and cortical thinning. However, this trajectory was found to be related to educational history. As such, the trajectory of an individual’s brain network composition has been proposed as a unique indicator of brain health in old age (Chan et al., 2021). Since the development of software to analyze brain networks using electroencephalography (EEG) (Pascual-Marqui et al., 2011), it has become possible to easily capture brain networks, such as the DAN and VAN, using rs–EEG (Aoki et al., 2023; Caravaglios et al., 2023).

Herein, we tested whether DAN and VAN could serve as direct neurophysiological markers for CR. DAN and VAN were measured using an EEG, a technique suitable for assessing the neural implementation of CR in community–dwelling older adults because it is non-invasive, relatively inexpensive, has few restrictions on the measurement location, and does not require a special license for measurement. Herein, we derived the DAN and VAN activity levels from rs–EEG, which have been shown to begin to change in patients with MCI, based on EEG measured at rest (Aoki et al., 2023; Caravaglios et al., 2023), and further tested whether they moderate the relationship between hippocampal volume and episodic memory performance. If so, it would suggest that specific EEG measures could be considered as neurophysiological markers of CR. If this were the case, these easily measured EEG markers could also help to determine the effectiveness of intervention studies aimed at preventing dementia by increasing CR.

## 2 Materials and methods

### 2.1 Study design

This investigation represents part of an ongoing study by the National Center for Geriatrics and Gerontology–Study of Geriatric Syndromes (NCGG–SGS) (Shimada et al., 2022a) to investigate health promotion for older adults in Aichi prefecture in Japan. The NCGG–SGS is a cohort study aimed at establishing a screening system for geriatric syndromes and validating evidence–based interventions to prevent them.

### 2.2 Standard protocol approvals, registrations, and participant consents

The research protocol was approved by the ethics committee of the National Center for Geriatrics and Gerontology (Approval Number: 1440–6). This study adhered to the principles outlined in the Declaration of Helsinki. All participants provided written informed consent prior to study inclusion.

### 2.3 Participants

**FIGURE 1:**
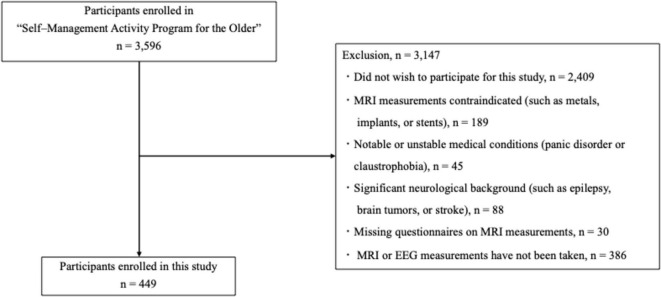
Flow diagram of sample selection.

Our inclusion criteria were as follows: a total of 449 participants (249 women; mean age: 74.0 years, standard deviation [SD]: 5.5 years; age range: 65–87 years) were recruited from the “Self–Management Activity Program for the Older” study (Shimada et al., 2022b). Exclusion criteria were as follows: (1) did not wish to participate for this study (n = 2409); (2) MRI measurements contraindicated (such as metals, implants, or stents) (n = 189); (3) notable or unstable medical conditions (panic disorder or claustrophobia) (n = 45); (4) significant neurological background (such as epilepsy, brain tumors, or stroke) (n = 88); (5) missing questionnaires on MRI measurements (n = 30); and (6) MRI or EEG measurements have not been taken (n = 386). Of the initial 3,596 participants, 3,147 were excluded based on these criteria (see Figure 1). The participants’ global cognitive function was evaluated using the Mini–Mental State Examination (MMSE) (Folstein et al., 1975). The measures described in the following sections were assessed in all participants.

### 2.4 Episodic memory assessments

Cognitive function was assessed using the National Center for Geriatrics and Gerontology Functional Assessment Tool (NCGG–FAT) (Makizako et al., 2013). Episodic memory was assessed using word-list memories I [immediate recognition] and II [delayed recall]. Immediate recognition and delayed recall involved the processing of a 10–word target list. To assess immediate recognition, participants were instructed to memorize 10 words, each shown for 2s on a tablet PC. Afterwards, a total of 30 words, including 10 target and 20 distracter words, were shown, and the participants were asked to identify the 10 target words (word list memory I). This procedure was repeated thrice. The mean number of correct answers was calculated using scores ranging from 0 to 10. Additionally, participants were instructed to recall (write down) the 10 target words after approximately 20 min (word list memory II). The total number of recalled target words was then recorded. One point was given for each correctly recalled word completed within 60 seconds, with a maximum score of 10 (Makizako et al., 2013). We subsequently calculated the episodic memory composite score using the sum of the immediate and delayed recognition scores (score range: 0–20) (Bae et al., 2020).

### 2.5 MRI acquisition and image processing and analysis

MRI was performed using a 3T Siemens MAGNETOM Trio Tim scanner (Siemens Medical Solutions, Erlangen, Germany), equipped with a 12-channel head coil. A three-dimensional T1-weighted magnetization prepared rapid acquisition gradient echo sequence was obtained in the sagittal plane with the following parameters: repetition time = 1800 ms, echo time = 1.99 ms, flip angle = 9°, 160 slices with a thickness of 1.1 mm, voxel size = 1.0 × 1.0 × 1.1 mm, image matrix = 256 × 256 mm, and field of view = 250 mm. The scans lasted for 4 min and 6 s each. Image processing was performed using FreeSurfer version 7^1^ on a Linux server running Ubuntu version 20.04 (Fischl et al., 2002). The automated processing pipeline involves several steps, including elimination of non-brain tissues, Talairach transformation, segmentation of gray and white matter tissues, intensity normalization, correction of cortical surface topology, and deformation of surfaces to enhance the accuracy of tissue boundary placement. The brain volumes (mm^3^) of the left and right hippocampi were computed from this data. Subsequently, to normalize the hippocampal volume, we divided the combined volume of the left and right hippocampi by the estimated total intracranial volume.

### 2.6 rs–EEG recording and analysis

The participants underwent rs–EEG recordings for 5 min, during which they were instructed to keep their eyes closed while remaining awake throughout the recording. Spontaneous cortical electrical activity was measured using a high-quality mobile dry-based 19-channel EEG system (CGX Quick-20r; Cognionics Inc.) and sampled at a rate of 500 Hz. EEG signals were acquired with electrodes placed according to the International 10–20 system (specifically, Fp1, Fp2, F3, F4, C3, C4, P3, P4, O1, O2, F7, F8, T3, T4, T5, T6, Fz, Cz, and Pz) using an ear reference. Electrode impedances were maintained below 10 kΩ. Bandpass filtering ranging from 0.53 to 120 Hz with a 60 Hz notch filter were applied using Brain Vision Analyzer software 2.2 (Brain Products, Munich, Germany).

We investigated the EEG data using exact low–resolution brain electromagnetic tomography (eLORETA), an open–source academic software available at http://www.uzh.ch/keyinst/loreta.htm (Pascual-Marqui et al., 2011). The eLORETA technique is used to estimate the cortical electrical patterns from scalp electrical potentials recorded at each electrode site. It can accurately identify any focal source within the brain by employing specific weights in a weighted minimum-norm inverse solution. While arbitrary distributions can be localized with reasonable accuracy based on the principles of linearity and superposition, the current iteration of eLORETA utilizes 6239 cortical gray matter voxels, with a spatial resolution of 5 mm within a realistic head model (Fuchs et al., 2002). The lead field was finally calculated based on anatomical labels corresponding to Brodmann areas. eLORETA uses a realistic head model to estimate the focal source, and it is widely used in a variety of EEG data research papers (Hata et al., 2016; Piano et al., 2017; Ikeda et al., 2019). The frequency bands under investigation (delta [2–4 Hz], theta [4–8 Hz], alpha [8–13 Hz], beta [13–30 Hz], and gamma [30–60 Hz]) aligned with those used in previous studies (Aoki et al., 2015, 2019; Katayama et al., 2023). Neural activity was calculated as the global field power value (Pascual-Marqui, 2002).

To identify spectral components that were maximally spatially independent, we conducted eLORETA–independent component analysis (ICA) on the eLORETA localization images, following the method outlined by Aoki et al. (2015, 2023), which is available in the eLORETA software. The eLORETA–ICA method decomposes non–Gaussian cortical electrical activity into independent components (ICs) across various frequency bands. It is a superior option for EEG data compared to alternative decomposition methods such as principal component analysis or correlation analysis (Bell and Sejnowski, 1997; Hyvärinen and Oja, 2000). eLORETA–ICA is capable of independently analyzing EEG activity in different frequency bands, allowing simultaneous and unambiguous identification of signals in multiple frequency bands (Aoki et al., 2015; Caravaglios et al., 2023). Conventional structural source localization methods, on the other hand, focus primarily on spatial resolution and are limited in their ability to discriminate frequency bands. We used eLORETA’s realistic head model because we want to apply our analysis method to future CR measurements in community–dwelling older adults who do not have MRI measures available. Therefore, we decided to use eLORETA’s realistic head model instead of estimating the focal source of EEG data by modeling individual anatomical structures from MRI measurements. The technical details of the eLORETA–ICA have been previously detailed by Pascual-Marqui et al. (2011). The mean localization image was computed for each participant across different frequency bands using the respective data that were subsequently concatenated. To discern a set of maximally independent components within the eLORETA spectrocortical electrical activity across a cohort of subjects, we employed group ICA using eLORETA–ICA software (Pascual-Marqui and Biscay-Lirio, 2011). The data matrix comprised subjects × (concatenated frequency bands and spatial dimensions [cortical voxels]). More precisely, the 5–frequency (delta, theta, alpha, beta, and gamma) source images derived from eLORETA for each subject were represented in a voxel-by-frequency matrix format, denoted as Nv × Nf, where Nv = 6239 (total number of voxels generated by eLORETA) and Nf = 5 (total number of frequency bands). ICA was subsequently applied to this data matrix to identify the maximally independent spectrocortical components (Cardoso, 1989; Cichocki and Amari, 2002). The ICs were subsequently arranged based on their total power and depicted using a color coding system for each frequency band. In this color-coded map, red and blue denote an increase and decrease in power, respectively, corresponding to enhanced IC activity. It is crucial to understand that ICA comprises two components: spectrocortical networks shared across all subjects and a set of “loadings” (i.e., network activities) unique to each subject. For an individual subject, these loadings (i.e., network activities) were used to quantify the contribution of each network to their specific spectrocortical activity. Furthermore, after identification of the spectrocortical networks common across a broad sample of subjects, these can be applied to the activity of any new subject, thereby generating loadings (i.e., network activities) specific to that individual (Aoki et al., 2019). That is, we used network activities as IC loading in the regression equation as follows: word list memory ∼ IC loading + covariates + interactions.

### 2.7 Confounding factors

Variables with a potential impact on episodic memory performance include demographic characteristics, chronic diseases, physical function, depressive symptoms, and living conditions associated with cognitive decline in older adults (Livingston et al., 2020; Collyer et al., 2022). As such, our multiple regression model included the following covariates: age, sex, years of education, heart disease, diabetes, hypertension, hyperlipidemia, number of medications, walking speed, 15-item Geriatric Depression Scale (GDS) score (Yesavage, 1988), living alone, and work status. Information on chronic diseases was obtained by a qualified nurse through a face–to–face interview with the participants. GDS scores and information on living alone and work status were assessed in face–to–face interviews with trained study assistants.

### 2.8 Statistical analysis

First, we tested the effect of hippocampal volume on memory performance using multiple regression analysis (“Main Effects”). Each multiple regression model included word list memory as the dependent variable and hippocampal volume as the independent variable, and was adjusted for age, sex, years of education, heart disease, diabetes, hypertension, hyperlipidemia, number of medications, walking speed, GDS score, living alone, and work status as covariates. The DAN and VAN network activities were subsequently evaluated in the same model after replacing the hippocampal volume with the DAN and VAN network activity. We evaluated the interactions between hippocampal volume and DAN and VAN network activity by adding the hippocampal volume x DAN and VAN network activity Interaction Terms in the model (“Interaction Terms”). For analyses where significant hippocampal volume × DAN and VAN network activity interaction (*p* < 0.05) was noted, we performed a *post-hoc* analysis using the Johnson–Neyman (JN) method to quantify the moderating effects of these network activities on the relationship between hippocampal volume and word list memory, as well as to identify the relevant ranges of DAN and VAN network activity (Chou et al., 2023). Overall, we identified a significant hippocampal–memory association. Finally, Spearman’s rank correlation coefficient was used to calculate the correlation coefficient between DAN and VAN activities. For all analyses, the significance level was set at *p* < 0.05. All analyses were performed using R version 4.2.2 (R Foundation for Statistical Computing, Vienna, Austria).

## 3 Results

### 3.1 Association between hippocampal volume and word list memory performance

Table 1 presents the demographic information of the participants. Larger hippocampal volume was associated with better memory performance (β = 0.154 (95% CI: 0.055; 0.253, *p* = 0.002, see Table 1).

**TABLE 1:** The demographic information of the participants.

Variable	Total (*n* = 449)
Age, median (IQR) [in year]	73 (69–78)
Sex, woman, *n* (%)	249 (55.5)
Education, median (IQR) [in year]	12 (12–16)
MMSE score, median (IQR)	29 (27–30)
Heart disease, yes, *n* (%)	57 (12.7)
Diabetes, yes, *n* (%)	64 (14.3)
Hypertension, yes, *n* (%)	170 (37.9)
Hyperlipidemia, yes, *n* (%)	157 (35.0)
Medication, median (IQR)	2 (1–4)
Walking speed, median (IQR) [in m/sec]	1.24 (1.12–1.36)
GDS score, median (IQR)	2 (1–4)
Living alone, yes, *n* (%)	76 (16.9)
Work, yes, *n* (%)	101 (22.5)
Word list memory composite score, median (IQR)	12.3 (9.7–14.3)
Hippocampal volume, median (IQR) [in % eTIV]	0.56 (0.52–0.60)
DAN, median (IQR) [in μV^2^/M^4^/Hz]	2566.4 (2225.5–3059.0)
VAN, median (IQR) [in μV^2^/M^4^/Hz]	9691.6 (9257.0–10072.2)

IQR, interquartile range; *n*, Number; MMSE, mini-mental state examination; GDS, 15–item geriatric depression scale; DAN, dorsal attention network; VAN, ventral attention network; eTIV, estimated total intracranial volume.

### 3.2 eLORETA–ICA

**FIGURE 2:**
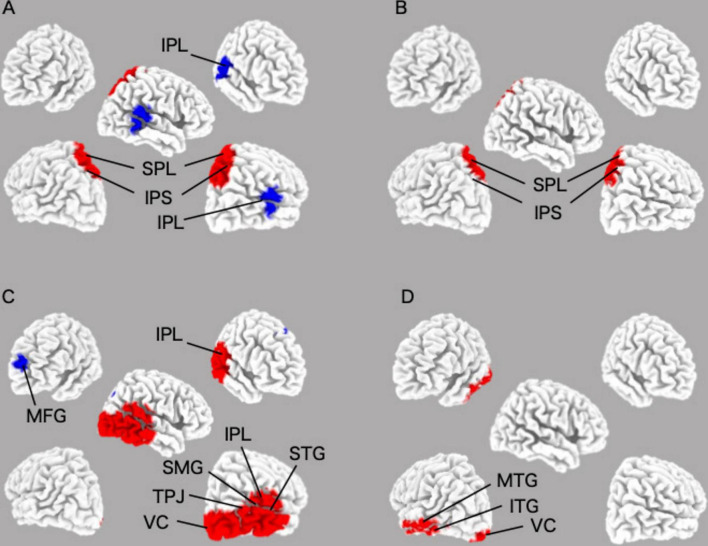
Images of the DAN and the VAN in their specified frequency bands were obtained by applying eLORETA–ICA to the EEG data. **(A)** beta of the DAN image; **(B)** gamma of the DAN image; **(C)** beta of the VAN image; **(D)** gamma of the VAN image. DAN, dorsal attentional network; VAN, Ventral attentional network.

Using eLORETA–ICA to analyze the rs–EEG data of 449 participants, we found that the number of independent components varied from 11 to 15. eLORETA–ICA does not calculate objective criteria or similarity measures to quantify the goodness of fit. In other words, there is no automatic matching to a template, based on spatial correlation or overlap. Therefore, IC selection was performed visually based on spatial maps, as in previous studies using eLORETA–ICA (Aoki et al., 2015, 2019; Jancke and Alahmadi, 2016; Caravaglios et al., 2023). This technique has been used in studies of functional brain networks using independent component analysis. Based on previous literature (Corbetta and Shulman, 2002; Corbetta et al., 2008; Tosoni et al., 2023), we selected ICs corresponding to DAN (IC–14) and VAN (IC–15) from a spatial map of ICs. To ensure the highest possible reliability of the visual inspection, we also checked whether the brain regions reported to contribute to VAN and DAN were included in the corresponding ICs. In addition, we led three researchers independently select the ICs for DAN and VAN and compared their level of agreement. Where there were differences in the selected ICs, they were discussed to reach consensus among the 3 researchers. Furthermore, we confirmed that the selected ICs were consistent with the functional properties of DAN and VAN. Three ICs turned out to represent artifact activities (IC–1, IC–4, and IC–12). These artifacts included an occipital baseline shift at the occipital cortex in the delta frequency band (IC–1 and IC–4) and an electromyogram at the temporal cortex in the gamma frequency band (IC–12) (Aoki et al., 2015). The mean values of DAN and VAN activity were 2727.5 ± 743.9 μV^2^/M^4^/Hz and 9618.3 ± 734.4 μV^2^/M^4^/Hz, respectively. The coefficient (Rs) and P value (p) of the correlation between DAN and VAN activities was Rs = −0.79, *p* < 0.001. The DAN consisted of bilateral beta and gamma activity in the bilateral superior parietal lobes and anti-correlated beta activity in the right temporoparietal junction, while the VAN consisted of beta activity in the right occipital to inferior parietal lobes and gamma activity in the left occipital and left inferior temporal lobes (Figure 2 and Table 2).

**TABLE 2:** The coordinates of DAN and VAN regions.

Network	IC	Frequency band	X (MNI)	Y (MNI)	Z (MNI)	Brodmann area	Structure
DAN	14	Beta	20	−65	65	7	SPL
DAN	14	Beta	−20	−60	65	7	SPL
DAN	14	Beta	65	−35	25	40	IPL
DAN	14	Gamma	15	−65	65	7	SPL
DAN	14	Gamma	−15	−60	65	7	SPL
VAN	15	Beta	−20	60	25	10	MFG
VAN	15	Beta	70	−30	5	42	STG
VAN	15	Beta	35	−65	40	39	IPL
VAN	15	Beta	65	−50	25	40	SMG
VAN	15	Beta	65	−50	20	22	TPJ
VAN	15	Beta	45	−85	−10	19	VC
VAN	15	Gamma	−65	−20	−15	21	MTG
VAN	15	Gamma	−60	−10	−25	20	ITG
VAN	15	Gamma	−10	−100	−5	17	VC

DAN, dorsal attention network; VAN, ventral attention network; ICs, independent components; SPL, superior parietal lobule; IPL, Inferior parietal lobule; MFG, middle frontal gyrus; STG, superior temporal gyrus; SMG, supramarginal gyrus; TPJ, temporoparietal junction; VC, visual cortex; MTG, middle temporal gyrus; ITG, inferior temporal gyrus.

### 3.3 DAN and VAN as moderators of the association between hippocampal volume and word list memory performance

Overall, we found a significant moderation of DAN on the hippocampus-memory association with a slope of β = −0.00012 (95% CI: −0.00024; −0.00001, *p* = 0.040, see Table 3) and of VAN with a slope of β = 0.00014 (95% CI: 0.00001; 0.00026, *p* = 0.031, see Table 3). Overall, we found no significant main effect of DAN or VAN, whereas the main effect of hippocampal volume remained significant in both models.

**TABLE 3:** Main and interaction effects of hippocampal volume and attentional networks.

	Word list memory		
Independent variables	β	95%CI	*p*
**Model 1:**			
Hippocampal volume	0.1543806	[0.0553502, 0.2534111]	0.002
**Model 2:**			
DAN activity values	0.0000029	[−0.0000045, 0.0000103]	0.439
**Model 3:**			
VAN activity values	−0.0000035	[−0.0000110, 0.0000040]	0.357
**Model 4:**			
Hippocampal volume	0.2009565	[0.0915488, 0.3103643]	< 0.001
DAN activity values	−0.0000024	[−0.0000160, 0.0000113]	0.735
VAN activity values	−0.0000012	[−0.0000149, 0.0000125]	0.863
Hippocampal volume x DAN	0.0000043	[−0.0002294, 0.0002380]	0.971
Hippocampal volume x VAN	0.0000374	[−0.0002098, 0.0002846]	0.767
DAN x VAN	−0.000000004	[−0.000000010, 0.000000002]	0.222
Hippocampal volume x DAN x VAN	0.0000001	[−0.000000003, 0.000000219]	0.057
**Model 5**			
Hippocampal volume	0.1552881	[0.0559102, 0.2546660]	0.002
DAN activity values	0.0000023	[−0.0000051, 0.0000096]	0.544
Hippocampal volume x DAN	−0.0001227	[−0.0002396, −0.0000058]	0.040
**Model 6:**			
Hippocampal volume	0.1525955	[0.0536656, 0.2515255]	0.003
VAN activity values	−0.0000029	[−0.0000103, 0.0000045]	0.441
Hippocampal volume x VAN	0.0001360	[0.0000130, 0.0002591]	0.031
**Model 7:**			
DAN activity values	−0.0000017	[−0.0000154, 0.0000121]	0.813
VAN activity values	−0.0000018	[−0.0000156, 0.0000120]	0.800
DAN x VAN	−0.000000003	[−0.000000009, 0.000000003]	0.324

DAN, dorsal attention network; VAN, ventral attention network; β: standardized regression coefficient; CI, confidence interval; *p*: *p*-value.

**FIGURE 3:**
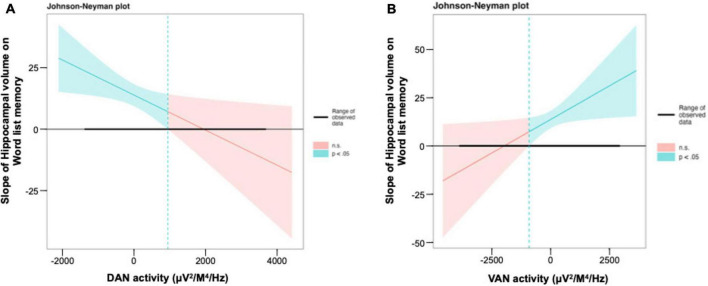
Interaction between the hippocampal volume and the DAN and the VAN for word list memory in regression model. **(A,B)** The Johnson–Neyman plot indicates the size and significance of the slope of hippocampal volume on word list memory throughout all observed levels of the DAN and the VAN activity. DAN, dorsal attention network; VAN, ventral attention network.

The JN plot shows the size and significance of the slope of the hippocampal volume on memory performance for all observed levels of the moderator variables, DAN (see Figure 3A), and VAN (see Figure 3B) activity. Overall, the larger the hippocampal volume, the better the memory performance. However, once DAN activity exceeded 944.9, hippocampal volume was no longer significantly associated with word list memory capacity (see Figure 3A). For VAN, hippocampal volume was no longer significantly associated with word list memory when VAN activity was less than −914.6 (Figure 3B).

## 4 Discussion

Overall, in the present study, we found a significant association between the hippocampal volume and word-list memory performance, which is consistent with the results of previous studies (Laakso et al., 2000; Walhovd et al., 2004). Further, the DAN and VAN activities detected by eLORETA–ICA were found to moderate the relationship between hippocampal volume and word-list memory performance. In particular, a high DAN activity and low VAN activity at rest supported the maintenance of episodic memory function in the presence of a lower hippocampal volume, as they were associated with a higher degree of independence of memory performance from hippocampal volume. Therefore, these measures may serve as neurophysiological markers of CR.

Beta and gamma activities observed in the DAN and VAN have been suggested to play important roles in attention, working memory, and long–term memory (Jensen et al., 2007; Marco-Pallarés et al., 2015). It has further been suggested that DAN activity is related to memory performance (Kragel and Polyn, 2015); however, we added the novel observation that DAN and VAN activity moderate the impact of lower hippocampal volume on memory performance. In a recent report, functional connectivity has been identified between the hippocampus and the DAN and VAN networks. It has been reported that these networks may modulate the hippocampus to switch between external and internal attention (Poskanzer and Aly, 2023). Therefore, it is possible that DAN and VAN activity may moderate the effect of hippocampal volume on word list memory. However, since this study is only a cross-sectional analysis, reverse causality cannot be ruled out. Activity in the DAN is believed to support the selection of sensory stimuli based on internal goals or expectations (goal–driven attention), as well as to link selected sensory stimuli to appropriate motor responses (Corbetta et al., 2008). The VAN works in the opposite manner to the DAN, primarily leading the detection of salient and behaviorally relevant stimuli in the environment (stimulus–driven attention) (Corbetta et al., 2008). Indeed, one rs–fMRI study confirmed that VAN connectivity is increased in the inattentive subtype of attention-deficit hyperactivity disorder (Sanefuji et al., 2017). It has further been reported that DAN and VAN interact dynamically to control information processing (Suo et al., 2021). Specifically, the role of DAN as a “network gate” that facilitates top–down attention processing by suppressing VAN and eliminating irrelevant bottom–up information has been identified (Pini et al., 2022). It has further been shown that the attention network of patients with amnestic MCI is selectively degenerated; specifically, functional connectivity is reduced in the DAN, whereas functional connectivity is maintained or enhanced in the VAN (Qian et al., 2015; Zhang et al., 2015). It has also been suggested that DAN plays an important role in facilitating top–down processes that suppress irrelevant information, that is, VAN control is diminished in AD (Pini et al., 2022). In this study, there was a significant strong negative correlation between DAN and VAN activities. However, the negative correlation between DAN and VAN activity values is only the result of the correlation analysis between the summarized 5-minute activities and does not directly imply an antiphase relationship observed in real time between the two networks. In other words, mean component activities of DAN and VAN activities are negatively correlated in this study, but it is not clear that real-time activity within subjects is anticorrelated. In future work, we would like to further clarify the dynamic relationship between attentional networks by examining the time course of DAN and VAN activities over the entire rs–EEG time course, to calculate the antiphase between the time courses of both networks in each time window.

The results of examining the significant range of the interaction using JN showed that while the main effect of hippocampal volume on memory performance was significant within a certain range for DAN, it became non-significant as DAN activity increased. Conversely, for VAN, the main effect of hippocampal volume on memory performance became non-significant as the VAN activity decreased. Overall, the JN technique is not useful in certain situations where the significance regions are small or the confidence intervals are so wide as to become practically useless, as, in such cases, the error variance is too large and/or the sample sizes are insufficient to provide adequate information (Potthoff, 1964). The simplest procedure for investigating the significance of interactions is the pick-a-point (Rogosa, 1980) or simple slope (Aiken et al., 1991) method. However, these methods select arbitrary values for the moderator variable, yielding information only for these arbitrary points (Carden et al., 2017). As such, the use of the more complex JN technique may provide a better resolution for clarifying interactions than traditional techniques, given that this technique examines significance along the continuum of values of the moderator, and delineates the slope of the relationship across each value (Garcia-Hermoso et al., 2021). Overall, these findings suggest that the activity patterns of high DAN and low VAN activity, or attentional networks, help to maintain memory performance in the presence of a lower hippocampal volume, as they make memory performance more independent of hippocampal atrophy, and may contribute to future research on dementia prevention.

Our findings are in line with those of previous studies reporting that IQ and education, traditionally treated as proxy markers for CR, are positively associated with DAN activity (Franzmeier et al., 2018; Koyama et al., 2020). Furthermore, DAN and VAN activities have been found to be associated with global cognitive function (Wang et al., 2015; Mao et al., 2020). It is therefore conceivable that high CR (high IQ and education levels) leads to increased DAN and decreased VAN activity, and that this activation pattern is associated with better memory performance and global cognitive function. It has further been suggested that rs–EEG activity differs across educational levels, with more-educated individuals exhibiting greater neuroprotective activity (Babiloni et al., 2020). Our results extend these findings by subjecting activity in attentional networks directly to the rigorous CR test suggested by the Reserve and Resilience Framework (Stern et al., 2020), and determining the optimal range for the moderation effect. These results further suggest that the attentional network is related to the neurophysiological background of reported CR proxy markers. As such, EEG measurement, which is non-invasive, relatively inexpensive, and allows measurements with minimal spatial constraints in community–dwelling older adults, has the potential to contribute to the assessment of CR. In the future, it would be exciting to examine the longitudinal changes in CR using rs–EEG, which is highly feasible in large cohort studies. The activity levels of the DAN and VAN may represent the neural implementations of the CR, which may help explain the results of intervention studies aimed at preventing dementia by increasing CR in terms of DAN and VAN activity.

This study had several limitations. First, the interaction effects of hippocampal volume with DAN and VAN are both significant but small, potentially limiting the generalizability and clinical relevance of the results. In addition, for the generalizability and clinical relevance of the results, further investigation of source estimation using anatomical structural data from each individual’s MRI images would be useful. Modulatory effects of transcranial magnetic stimulation (TMS) and repeated transcranial direct current stimulation (tDCS) on attentional networks have been reported (Sacca et al., 2023; Massironi et al., 2024). As such, it may be useful to conduct future studies to clarify whether modulating the DAN and VAN through interventions, such as TMS and tDCS, are effective at maintaining good memory performance in the presence of age-related brain pathologies.

Next, the DAN activity is inversely correlated with the default mode network (DMN) activity, and a growing number of reports have indicated that this inverse correlation is related to cognitive function (Kelly et al., 2008; Franzmeier et al., 2017; Wang et al., 2019). Therefore, further studies are needed to disentangle the contributions of each brain network by including the activity of the DMN together with the activity of the DAN and VAN. In addition, this cross–sectional study did not examine the association between longitudinal changes in cognitive function. To address this limitation, a 30–month follow–up examination is currently underway to examine the relevance of baseline attentional network activity to longitudinal changes in the relationship between hippocampal volume and episodic memory performance. Finally, this study focused solely on the relationship between the hippocampal volume and episodic memory performance. Therefore, it would be of interest to test whether DAN and VAN network activities moderate the association between other brain regions and cognitive performance.

## 5 Conclusion

Herein, we show that the noninvasive and relatively inexpensive eLORETA–ICA approach for analyzing rs–EEG data may be suitable for capturing the neural implementation of CR. rs–EEG measurement is easy to perform and is not limited by the measurement environment. Our study suggests that CR can be measured in community–dwelling older adults using this technique. Neurophysiological markers of CR measured by rs–EEG may help to identify individuals at high risk of developing dementia, as well as to monitor the efficiency of specific interventions to promote attention network activity, such as TMS and tDCS, which has been reported to modulate DAN and VAN activity.

## Data availability statement

The raw data supporting the conclusions of this article will be made available by the authors, without undue reservation.

## Ethics statement

The studies involving humans were approved by the Ethics Committee of the National Centre for Geriatrics and Gerontology. The studies were conducted in accordance with the local legislation and institutional requirements. The participants provided their written informed consent to participate in this study.

## Author contributions

OK: Conceptualization, Data curation, Formal analysis, Funding acquisition, Methodology, Validation, Visualization, Writing−original draft, Writing−review and editing. YS: Conceptualization, Methodology, Supervision, Writing−review and editing. CH: Conceptualization, Methodology, Supervision, Writing−review and editing. AC: Data curation, Methodology, Visualization, Writing−review and editing, Validation. SL: Conceptualization, Funding acquisition, Methodology, Writing−review and editing. KH: Conceptualization, Methodology, Writing−review and editing. KM: Conceptualization, Methodology, Writing−review and editing. KT: Conceptualization, Formal analysis, Methodology, Writing−review and editing. MM: Conceptualization, Formal analysis, Methodology, Writing−review and editing. RY: Conceptualization, Methodology, Writing−review and editing. CN: Conceptualization, Methodology, Writing−review and editing. YM: Conceptualization, Methodology, Writing−review and editing. KF: Conceptualization, Methodology, Writing−review and editing. TK: Conceptualization, Formal analysis, Funding acquisition, Methodology, Writing−review and editing. HS: Conceptualization, Funding acquisition, Methodology, Project administration, Resources, Supervision, Writing−review and editing.

## Footnotes

1
http://surfer.nmr.mgh.harvard.edu
